# Long non-coding RNA00364 represses hepatocellular carcinoma cell proliferation via modulating p-STAT3-IFIT2 signaling axis

**DOI:** 10.18632/oncotarget.22039

**Published:** 2017-10-25

**Authors:** Wei-Guo Tang, Bo Hu, Hai-Xiang Sun, Qi-Man Sun, Chao Sun, Pei-Yao Fu, Zhang-Fu Yang, Xin Zhang, Chen-Hao Zhou, Jia Fan, Ning Ren, Yang Xu

**Affiliations:** ^1^ Department of Liver Surgery, Liver Cancer Institute, Zhongshan Hospital, Fudan University; Key Laboratory of Carcinogenesis and Cancer Invasion, Ministry of Education, Shanghai 200032, P. R. China; ^2^ Department of Surgery, Minhang Branch of Zhongshan Hospital, Fudan University, Shanghai 201199, P. R. China; ^3^ Institute of Fudan-Minhang Academic Health System, Minhang Hospital, Fudan University, Shanghai 201199, P. R. China

**Keywords:** hepatocellular carcinoma, long noncoding RNA00364, interferon-γ, p-STAT3/IFIT2, prognosis

## Abstract

The effects of long non-coding RNAs (lncRNAs) on hepatocellular carcinoma (HCC) remain largely unclear. In this study, we identified an interferon (IFN)-γ-induced LncRNA, LncRNA00364, in HCC by microarray. LncRNA00364 displays lower expression in HCC tumor samples compared to paired normal controls. Overexpression of LncRNA00364 inhibits cell proliferation, G1/S cell cycle progression and promotes apoptosis in HCC cell lines. Consistently, LncRNA00364 overexpression leads to decreased HCC tumor formation *in vivo*. Mechanistically, LncRNA00364 specifically binds with STAT3, resulting in inhibition of STAT3 phosphorylation and therefore leads to upregulation of IFIT2. In a clinical setting, LncRNA00364 shows an independent prognostic indicator for overall survival and cumulative recurrence in HCC patients, and correlates with IFIT2. Therefore, our study provides new insights into a novel therapeutic avenue targeting the LncRNA00364 signaling axis in HCC.

## INTRODUCTION

Hepatocellular carcinoma (HCC) is the third most common cause for cancer mortality worldwide [1]. Genetic and environmental factors are closely linked to the development of HCC and over 700,000 patients die of HCC annually [2]. Surgical resection only provides a treatment option for patients that are diagnosed at an early stage. For patients with advanced HCC, the introduction of sorafenib, a small molecular inhibitor for several kinase inhibitors that target VEGFR, PDGFR and RAF family kinases, may offer limited survival benefits [3]. Despite the recent progress in HCC diagnosis and treatment for this refractory disease remains unsatisfactory due to dismal prognosis and frequent recurrence. Thus, identification of novel targets and better understanding the underlying mechanism are urgently needed for effective therapeutic intervention in HCC.

Long non-coding RNAs (lncRNAs) are RNA transcripts that are longer than 200bp with no apparent protein-coding ability [4]. Normally, lncRNAs show a low level of sequence conservation across different mammalian species. However, their secondary structures, which are crucial for their function, tend to be conserved. As mentioned above, lncRNAs are regulatory RNAs, which are transcribed by RNA polymerase II/III. In comparison to protein-coding genes, lncRNAs often show low level of expression and exhibit tissue-specific expression patterns [5, 6]. Most of expressing lncRNAs are localized in the nucleus and some of them are exported to the cytoplasm [7]. They participate in a wide range of biological processes, including development, differentiation, stem cell pluripotency and reprogramming [8, 9]. In addition, they play vital roles in pathological status diseases, including cancer.

Accumulating evidence suggests that lncRNAs can affect a plethora of cellular functions and participate in diverse physiological and pathological processes [10]. The aberrant expression of lncRNAs has been demonstrated in multiple malignancies, including HCC [11–16], which provides new insights into the pathogenesis of cancer.

It is well established that Interferon(IFN), especially the type II IFN such as IFN-γ, plays a very important role in anti-tumor immunity. IFN-γ has been reported to repress cell proliferation and promote apoptosis in many types of tumors, such as HCC [17], ovarian cancer [18], bladder cancer [19], pancreatic cancer [20] and melanoma [21]. The NeST Long ncRNA has been reported to regulate expression of IFN-γ in bacteria and viral pathogens [22]. However, the potential role of lncRNAs involving IFN-γ in tumorigenesis such as HCC remains largely unknown. Thus, there is an urgent need to elucidate the regulation of lncRNAs by IFN-γ in HCC, which is critical for the identification of new therapeutic targets and genetic biomarkers in this lethal cancer.

LncRNA00364, as key regulator involved in controlling fundamental tumorigenesis processes, locates in the chromosome 13q21.32. However, LncRNA00364 expression and its regulatory pathway have not been largely studied in HCC. In the present study, we demonstrate that LncRNA00364 is downregulated in HCC compared to normal, and its overexpression inhibits cell proliferation, promotes cell cycle arrest and apoptosis. We also show LncRNA00364 predominantly localizes in the cytoplasm of HCC cells. In this study, we find that STAT3 can inhibit transcription of IFIT2. The knockdown of IFIT2 results in decreased apoptosis *in vivo and vitro*. Furthermore, mechanistic studies demonstrate that LncRNA00364 inhibits STAT3 phosphorylation and upregulates the IFIT2 expression.

## RESULTS

### Expression level of LncRNA00364 downregulated in HCC

**Figure 1:**
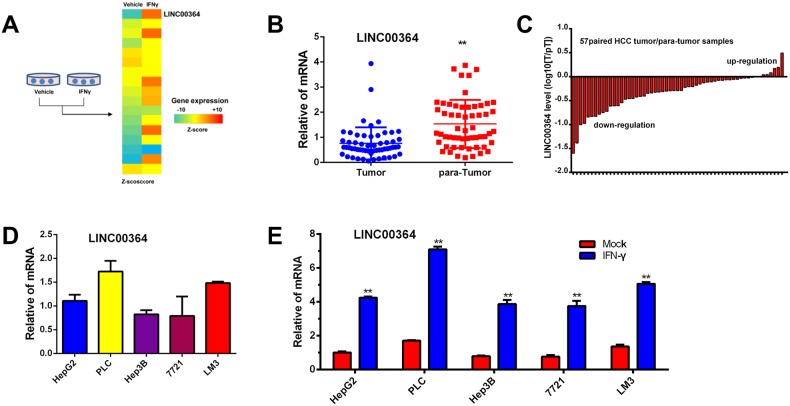
Interferon-γ stimulated leads to LncRNA00364 upregulation in HCC **(A)** Heat map showing all the upregulated genes including LncRNA00364 induced by interferon –γ treatment in HepG2 cells based on microarray analysis. **(B, C)** Relative LncRNA00364 expression in 57 paired human HCC tissues and para-tumor tissues by qRT-PCR analysis, ^**^p<0.01. **(D)** LncRNA00364 mRNA expression in HCC cell lines including HepG2, PLC, Hep3B, 7721 and LM3 by using qRT-PCR. **(E)** LncRNA00364 mRNA expression in HCC cell lines treated with mock control or IFN-γ. ^**^p<0.01. Results were triplicate experiments with mean ± SD. ^*^P<0.05, ^**^P<0.01.

In order to systematically examine the effect of immunotherapy drug IFN-γ on gene regulation in HCC, we performed gene expression profiling of HepG2 treated with IFN-γ and revealed that IFN-γ treatment affected a variety of gene expression. Among them, LncRNA00364 mRNA displays the most robust regulation and IFN-γ induces its expression level by more than two folds (Figure 1A and Supplementary Table 1). To determine the functional significance of LncRNA00364 in HCC patients, we further analyzed the level of LncRNA00364 in HCC specimens by performing quantitative RT-PCR assays on 57 paired hepatocellular carcinoma vs adjacent liver tissues. Notably, LncRNA00364 was found to be down-regulated in HCC tumor tissues compared to paired adjacent liver tissues (Figure 1B and 1C, p<0.01). To determine the role of LncRNA00364 in HCC development, we first examined the LncRNA00364 expression in commonly used HCC cell lines, HepG2, LM3, PLC, Hep3B and 7721 by quantitative RT-PCR assays. We found that LncRNA00364 had a relatively low expression in HepG2 cells, and relatively high expression in LM3 cells (Figure 1D), thus we chose these two cell lines in further studies. To validate our microarray data, we treated HepG2, LM3, PLC, Hep3B and 7721 cells with IFN-γ, and found that LncRNA00364 upregulated at the mRNA levels (p<0.01, Figure 1E). Collectively, these observations indicated that LncRNA00364 was down-regulated in HCC cell lines and tumor tissues of HCC patients.

### LncRNA00364 suppresses proliferation and promotes apoptosis of HCC *in vitro* and *in vivo*

**Figure 2:**
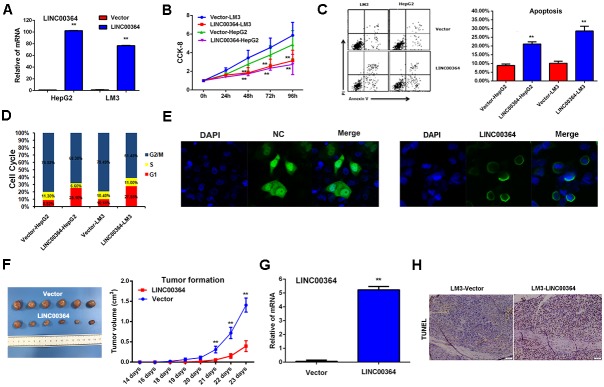
LncRNA00364 inhibits proliferation and promotes apoptosis of HCC cells *in vitro* and *in vivo* **(A)** The expression of LncRNA00364 in HepG2 and LM3 cell lines, ^**^p<0.01. **(B)** Cell proliferation detected by CCK-8 assay in each line as indicated, ^**^p<0.01. **(C)** Cell apoptosis detected by Annexin-V-FITC assay in each cell line as indicated, ^**^p<0.01. **(D)** Cell cycle detected by PI staining assay in each cell line as indicated. **(E)** RNA FISH analysis of LncRNA00364 localization in HepG2 cells. **(F)** The average volume of tumors between the LncRNA00364 overexpression and control groups from six independent experiments, ^**^p<0.01 (n=6) compared with vector group. **(G)** Expression of LncRNA00364 was analyzed in the tumor xenografts tissues from mice by qRT-PCR, ^*^p<0.01. **(H)** The apoptosis expression of the tumor xenografts tissues from mice was detected by TUNEL assay, ^**^p<0.01. Results were triplicate experiments with mean ± SD. ^*^P<0.05, ^**^P<0.01.

We firstly evaluated the effects of LncRNA00364 on cell proliferation, apoptosis and cell cycle, and then stably introduced lentiviral vectors containing cDNA encoding the full lenghLncRNA00364 into HCC cell line HepG2 and LM3 via viral infections, and performed qRT-PCR to confirm LncRNA00364 expression in these cells (p<0.01, Figure 2A). By using CCK-8 assays, we found stable expression of LncRNA00364 resulted in decreased cell growth (p<0.01, Figure 2B), increased apoptosis (p<0.01, Figure 2C), and a defect in G1/S transition in both HepG2 and LM3 cells (Figure 2D). In addition, we examined the cellular location of LncRNA00364 by using RNA FISH (fluorescent in situ hybridization), we found that LncRNA00364 displayed a predominant cytoplasmic localization in HepG2 cells (Figure 2E). To determine the effects of LncRNA00364 in HCC development *in vivo*, Consistently with our observations *in vitro*, overexpression of LncRNA00364 significantly decreased tumor growth and induced apoptosis over time compared to the vector control (Figure 2F, 2G and 2H).

### LncRNA00364 suppresses IFN-γ-stimulated STAT3 phosphorylation by directly interacting with STAT3

**Figure 3:**
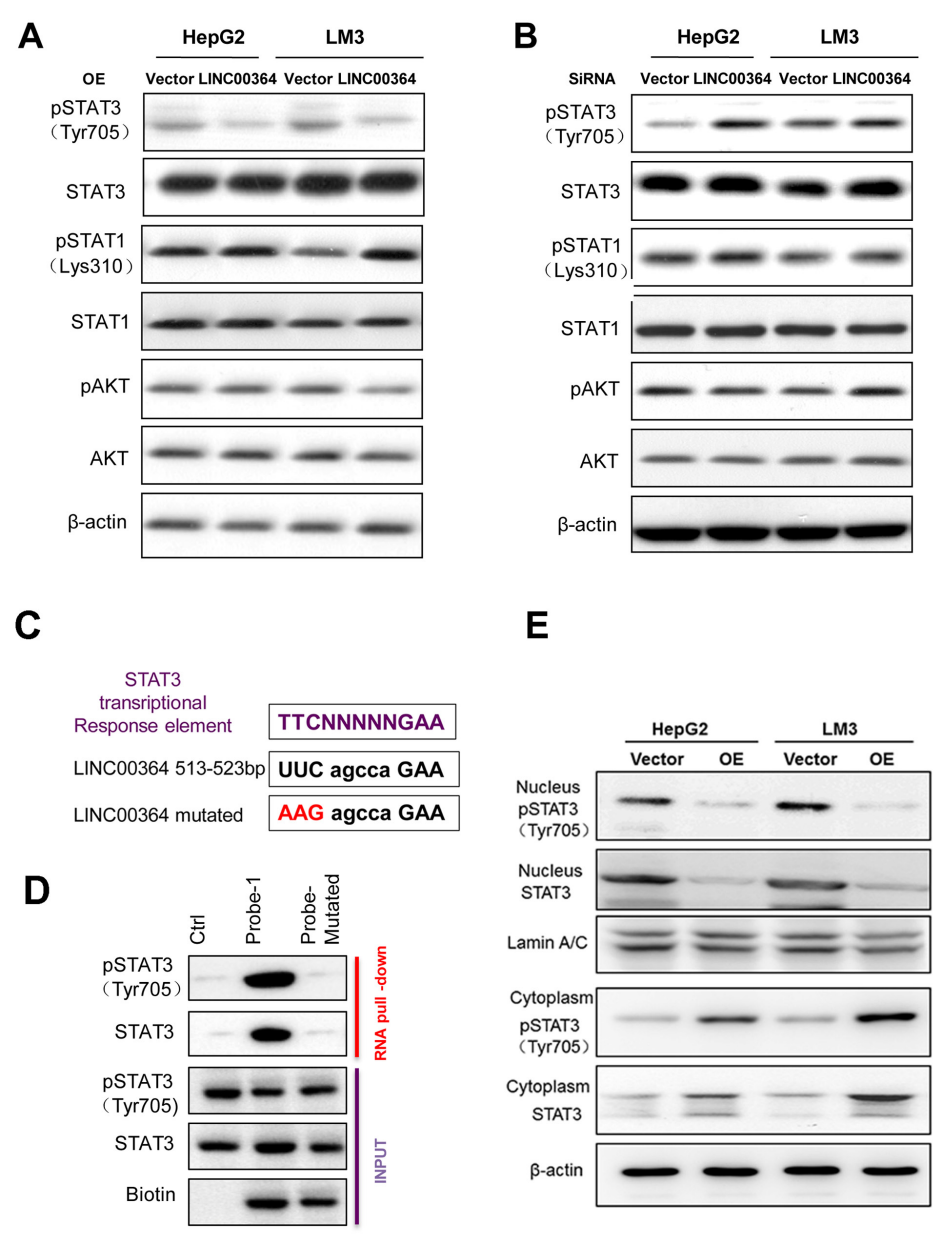
LncRNA00364 suppresses STAT3 phosphorylation on Tyr705 **(A, B)** The protein level of STAT3, pSTAT3 (Tyr705), STAT1, pSTAT1 (Lys310), AKT and pAKT with LncRNA00364 overexpression or deficiency were analyzed by western blot assays. **(C)** Diagram showing the conserved STAT3 transcriptional response element of LncRNA00364. **(D)** RNA pull-down analysis to determine the interaction of phosphorylated STAT3 with mutated LncRNA00364 in HepG2 cells. **(E)** The distribution of pSTAT3 and STAT3 in cytoplasm and nucleus in HCC cells transfected with LncRNA00364 overexpression or vector by nucleocytoplasmic separation.

To elucidate the molecular mechanism of LncRNA00364 in HCC, we examined the potential effect of LncRNA00364 on the JAK/STAT signaling given its important role in HCC [23–25]. Firstly, we examined the effects of LncRNA00364 on the phosphorylation status of STAT1, STAT3 and AKT by western blot in HepG2 and LM3 cells. Interestingly, LncRNA00364 overexpression led to decreased phosphorylation of STAT3 while not affecting STAT1 or AKT phosphorylation (Figure 3A). As a complementary approach, we depleted LncRNA00364 by using the validated specific siRNA and found that STAT3 phosphorylation is upregulated (Figure 3B and Supplementary Figure 1A).

Our data suggests that LncRNA00364 specifically inhibits STAT3 phosphorylation. The JAK1 mRNA expression was not apparently changed in cells overexpressing LncRNA00364 (Supplementary Figure 1B). To further analyze how LncRNA00364 regulates STAT3 phosphorylation, we identified an evolutionarily conserved STAT3 transcriptional response element in LncRNA00364(Figure 3C). Whereas the wild type LncRNA00364 probe could efficiently pull down STAT3 or phosphorylated STAT3 (Tyr705), the mutant probe that contains STAT3 response element mutations could not do so (Figure 3D), suggesting that LncRNA00364 can bind STAT3 directly. We further observed that LncRNA00364 overexpression inhibited the phosphorylation of STAT3 and its nuclear import in HepG2 and LM3 cells by nucleocytoplasmic separation (Figure 3E). Taken together, these data suggest that LncRNA00364 suppresses STAT3 phosphorylation by directly interacting with STAT3.

### LncRNA00364 inhibits proliferation through upregulation of IFIT2 *in vitro* and *in vivo*

**Figure 4:**
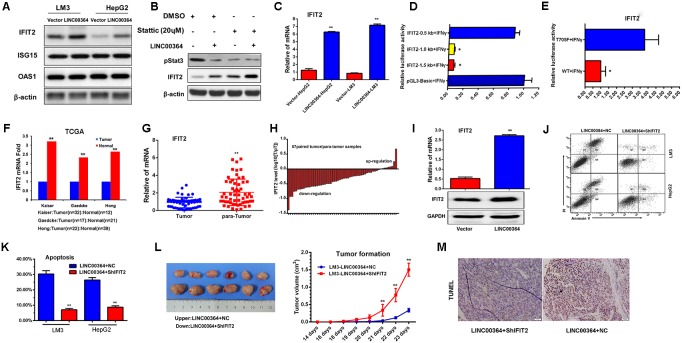
LncRNA00364 increases apoptosis through upregulation of IFIT2 *in vitro* and *in vivo* **(A)** The protein levels of IFIT2, ISG15 and OAS1 with LncRNA00364 overexpression analyzed by western blot assays. **(B)** The p-STAT3 and IFIT2 protein level with LncRNA00364 overexpression or STAT3 inhibitor Stattic analyzed by western blot assays. **(C)** The mRNA levels of IFIT2, ISG15 and OAS1 with ectopic expression analyzed by Real-time PCR assays. **(D)** Luciferase reporter assay (RLA) in 293T cells co-transfected with the different length of IFIT2 promoter reporter gene, STAT3 and Renilla 24 h, treated with interferon-γ for 12 h. RLA is shown as the mean of three independent experiments ± SD. ^*^P <0.05, IFIT2 1.0Kb and 1.5Kb group compared with pGL3-basic group. **(E)** Luciferase reporter assay (RLA) in 293T cells co-transfected with the IFIT2 promoter reporter gene, mutation of STAT3 or WT, and Renilla 24 h, treated with interferon-γ for 12 h. RLA is shown as the mean of three independent experiments ± SD. ^*^P <0.05, WT group compared with T705F group. **(F)** IFIT2 expression in datasets of Kaiser, Hong and Gaedcke from the Oncomine database. ^**^p<0.01. **(G)** Expression of IFIT2 in 57 paired human HCC tissues and para-tumor tissues compared by real-time PCR, ^**^p<0.01. **(H)** qRT-PCR analysis of IFIT2 expression in 57 paired HCC tumor tissues and adjacent normal tissues. **(I)** The IFIT2 mRNA and protein levels were detected in the tumors from mice transplanted with LM3 cells stably transfected with LINC00364 or vector, ^**^p<0.01. **(J)** Cell apoptosis detected in LM3-LINC00364 and HepG2-LINC00364 cells transfected with control or ShIFIT2 by Annexin-V-FITC assay. **(K)** Apoptosis rate shown in the histogram as indicated, ^**^p<0.01. **(L)** Tumor volume and tumor growth curve shown in the xenograft models of nude mice subcutaneously transplanted with LM3-LINC00364 cells stably transfected with control or ShIFIT2, respectively. ^**^p<0.01. **(M)** Apoptosis detected by TUNEL assay in the above xenograft models as indicated. Scale bar is 50 μm. ^**^p<0.01. Results were triplicate experiments with mean ± SD. ^*^P<0.05, ^**^P<0.01.

To identify the downstream targets of LncRNA00364 that mediate its phenotype on cell proliferation, we first examined the expression of the most commonly studied Interferon-stimulated genes(ISG) such as IFIT2, OAS1 and ISG15 to see if they were regulated by LncRNA00364-STAT3. Notably, overexpression of LncRNA00364 resulted in upregulated IFIT2 and downregulated p-STAT3 in HepG2 and LM3 cells (Figure 4A, 4B and 4C). while, STAT3 inhibitor Stattic could accelerate the upregulation of IFIT2 (Figure 4B). To further analyze how STAT3 regulates IFIT2, we transferred different length of IFIT2 promoter and STAT3 and Renilla (internal reference) in 293t cells, adding interferon treatment 12hrs by using the luciferase reporter system assay. The study found that 0.5kb can not be inhibited, and 1.0kb and 1.5kb can be significantly inhibited. The result showed that STAT3 inhibited IFTI2 mainly in 0.5 kb to 1.0 kb (Figure 4D). The experiment says that STAT3 can directly inhibit the transcription of IFIT2.

In the 293t cells, we further transferred the IFIT2 1.5kb promoter and STAT3 (705 mutation and wild type) and Renilla (internal reference), adding IFN-γ. The result shows that STAT3 inhibits the transcription of IFIT2 mainly through the T705 site, mutating out of this site can not inhibit transcription (Figure 4E). This is also consistent with our data (Figure 3A and 3B).

On the other hand, overexpression of LncRNA00364 fail to show any apparent effects on the expression of OAS1 and ISG15 (Figure 4A and Supplementary Figure 1C and 1D), suggesting its specific effect on IFIT2. IFIT2 is well known as an important inducer of apoptosis and therefore serves as an important tumor suppressor [26–28]. To examine the physiological relevance of IFIT2 in HCC, we examined its expression in normal and HCC patients in several independent datasets available through the oncomine database. Our data analyses showed that IFIT2 was downregulated in HCC tumors compared to normal patients (Figure 4F), which is consistent with LncRNA00364 expression pattern between HCC tumors and normal tissues. To strengthen our findings, we also performed real-time PCR assays on 57 paired HCC tumors and adjacent normal tissues (para-tumor). Consistent with previous results, the expression of IFIT2 was downregulated in tumor compared to para-tumor (p<0.01, Figure 4G and 4H). In xenograft tumors derived from cell lines that contain either control or LncRNA00364 overexpression vector, the expression of IFIT2 was examined by qRT-PCR and Western-blot assays. IFIT2 mRNA and protein levels were significantly higher in tumors formed from cells overexpressing LncRNA00364 compared to the vector controls (Figure 4I). To further evaluate the apoptosis promotion of IFIT2 *in vivo and vitro*, we knocked down IFIT2 and found that apoptosis decreased in the LM3-LINC00364 and HepG2-LINC00364 cells (p<0.01, Figure 4J and 4K) in vitro. Consistently with vitro, IFIT2 knockdown resulted in increased tumor growth (p<0.01, Figure 4L) and decreased apoptosis (p<0.01, Figure 4M) in tumors from nude mice transplanted with LM3-LINC00364-ShIFIT2 cells compared to control group. Taken together, these data demonstrate that IFIT2 may be served as a downstream target gene of LncRNA00364-STAT3 which mediating HCC proliferation *in vitro* and *vivo*.

### LncRNA00364 positively correlates with IFIT2 and its lower expression predicts worse prognosis in HCC patients

**Figure 5:**
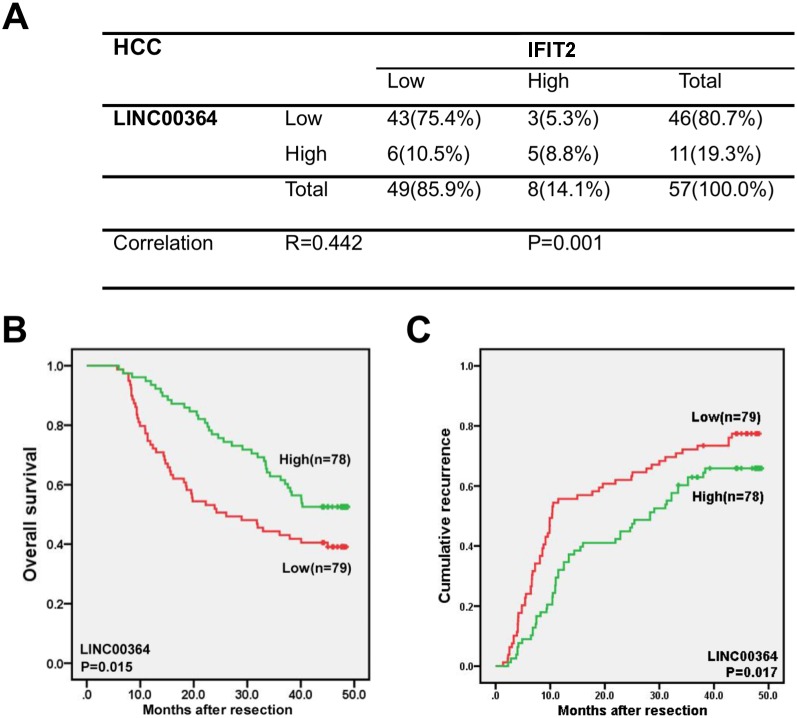
LncRNA00364 positively correlates with IFIT2 and its lower expression predicts worse patient prognosis in HCC **(A)** Significantly positive correlation between LncRNA00364 and IFIT2 in HCC samples. (^**^p<0.01, n=57). **(B, C)** The correlation between LncRNA00364 and OS, TTR in HCC samples. (OS ^*^p=0.015, n=157; TTR ^*^p=0.017, n=157).

To evaluate the clinical relevance of LncRNA00364 and IFIT2/ISG54, Real-time PCR assays were performed on 57 paired HCC specimens. Regression analysis showed that a significantly positive correlation between the level of LncRNA00364 and IFIT2 in HCC samples (Figure 5A, p<0.01).

We further explored the clinical implications of LncRNA00364 expression in HCC patients. The cohort of 157 HCC patients was divided into low and high expression groups according to the median of LncRNA00364 level in tumors. Our detailed analyses showed that LncRNA00364 level remarkably correlated with several tumor characteristics (Table 1), including tumor size (P= 0.001), tumor encapsulation (P = 0.027) and HBeAg expression (P=0.002).

**Table 1:** Correlation between the factors and clinicopathologic characteristics in HCC (n=157)

Clinicopathological indexes	Lncrna00364		
	Low	High	P
**Age(year)**	26	28	0.411
**≤50**	53	50	
**>50**			
**Sex**	9	16	0.132
**Female**	70	62	
**Male**			
**HBsAg**	6	9	0.285
**Negative**	69	73	
**Positive**			
**HBcAb**	44	38	0.237
**Negative**	35	40	
**Positive**			
**HBeAg**	75	61	0.002
**Negative**	4	17	
**Positive**			
**HBV-DNA**	50	49	0.541
**Negative**	29	29	
**Positive**			
**HCV**	76	76	0.506^*^
**Negative**	3	2	
**positive**			
**AFP (ng/ml)**	47	41	0.238
**≤20**	32	37	
**>20**			
**TB(umol/L)**	73	67	0.146
**≤20**	6	11	
**>20**			
**ALT(U/L)**	67	63	0.323
**≤50**	12	15	
**>50**			
**PALB(g/L)**	20	21	0.481
**≤0.25**	59	57	
**>0.25**			
**ALB(g/L)**	27	17	0.06
**≤35**	52	61	
**>35**			
**GGT (U/L)**	57	54	0.410
**≤60\**	22	24	
**>60**	22	24	
**PT(s)**	60	66	0.122
**≤13**	19	12	
**>13**			
**AKP(U/L)**	65	67	0.344
**≤125**	14	11	
**>125**			
**Liver cirrhosis**	31	39	0.116
**No**	48	39	
**Yes**			
**Tumor size(cm)**	26	45	0.001
**≤5**	53	33	
**>5**			
**Tumor number**	62	64	0.359
**Single**	17	14	
**Multiple**			
**Microvascular invasion**	29	27	0.457
**Absence**	50	51	
**Present**			
**Tumor encapsulation**	43	55	0.027
**Complete**	36	23	
**None**			
**Tumor differentiation**	41	43	0.403
**I+II**	38	35	
**III+IV**			
**TNM stage**	27	33	0.188
**I**	52	45	
**II+III**			

AFP, alpha-fetoprotein; GGT, gamma glutamyl transferase; TNM, tumor-node-metastasis; ALT, alanine transaminase;

TB, total bilirubin; PALB, prealbumin; ALB, albumin; AKP, alkaline phosphatase;

^*^Fisher's exact tests; chi-square tests for all other analyses.

We also found that the lower expression of LncRNA00364 was obviously correlated with decreased survival and increased risk for postoperative recurrence in HCC patients (Figure 5B and 5C). Patients in high LncRNA00364 expression group had significantly longer overall survival(OS) (Figure 5B, P=0.015) and time to recurrence(TTR) (Figure 5C, P=0.017) than those in low expression groups.

LncRNA00364 expression was confirmed as an independent predictor of prognosis for both OS (hazard ratio=0.546, 95% CI 0.333-0.895, *P*=0.016) and TTR (hazard ratio=0.576, 95% CI 0.379-0.876, P=0.017) by using univariable and multivariate analysis (Table 2).

**Table 2:** Univariate and multivariate analyses of prognostic factors in HCC (n=157)

Variable	TTR		OS	
	HR (95% CI)	P	HR (95% CI)	P
**Univariate analysis**				
Age, year (≤50 versus >50)	0.648(0.441-0.952)	0.027	0.587(0.382-0.903)	0.015
Sex (female versus male)	1.032(0.615-1.729)	0.906	1.149(0.624-2.116)	0.656
HBsAg (negative versus positive)	1.663(0.841-3.291)	0.144	2.936(1.075-8.022)	0.036
HBcAb (negative versus positive)	0.365(0.248-0.538)	0.000	0.230(0.141-0.376)	0.000
HBeAg (negative versus positive)	0.993(0.598-1.648)	0.977	0.422(0.195-0.916)	0.029
HBV-DNA(negative versus positive)	0.916(0.624-1.347)	0.657	0.903(0.578-1.408)	0.652
HCV-RNA(negative versus positive)	0.637(0.202-2.008)	0.442	0.277(0.039-1.990)	0.202
AFP, ng/ml (≤20 versus >20)	0.543(0.370-0.797)	0.002	0.476(0.302-0.749)	0.001
TB(umol/L) (≤20 versus >20)	2.147(1.238-3.726)	0.007	2.242(1.258-3.996)	0.006
ALB(g/L)( ≤35 versus >35)	0.537(0.36-0.799)	0.002	0.596(0.382-0.931)	0.023
ALT(U/L)( ≤50 versus >50)	1.313((0.822-2.096)	0.254	0.979(0.552-1.737)	0.943
PALB(g/L) (≤0.25 versus >0.25)	2.376(1.487-3.798)	0.000	5.251(2.530-10.898)	0.000
AKP(U/L)(≤125 versus >125)	1.263(0.778-2.050)	0.344	1.157(0.652-2.053)	0.618
PT(s) ( ≤13 versus >13)	1.191(0.763-1.858)	0.442	0.797(0.449-1.414)	0.438
GGT, U/L (≤60 versus >60)	0.846(0.563-1.270)	0.420	0.908(0.570-1.449)	0.678
Liver cirrhosis (no versus yes)	1.851(1.259-2.722)	0.002	2.148(1.365-3.381)	0.001
Tumor size, cm (≤5 versus >5)	1.378(0.946-2.007)	0.095	2.243(1.418-3.548)	0.001
Tumor number (single versus multiple)	1.574(1.007-2.460)	0.047	1.774(1.090-2.887)	0.021
Microvascular invasion (no versus yes)	1.971(1.306-2.974)	0.001	2.620(1.585-4.332)	0.000
Tumor encapsulation (complete versus none)	3.799(2.586-5.581)	0.000	5.214(3.358-8.095)	0.000
Tumor differentiation (I + II versus III + IV)	1.151(0.794-1.667)	0.457	0.861(0.561-1.322)	0.493
TNM stage (I versus II III)	3.225(2.102-4.947)	0.000	4.649(2.653-8.146)	0.000
LINC00364(low versus high)	0.639(0.440-0.927)	0.018	0.591(0.385-0.909)	0.017
**Multivariate analysis**				
Age, year (≤50 versus >50)	0.876(0.565-1.359)	0.555	0.854(0.530-1.377)	0.517
HBcAb (negative versus positive)	0.466(0.247-0.880)	0.019	0.379(0.184-0.781)	0.008
TB(umol/L) (≤20 versus >20)	1.050(0.541-2.039)	0.885	0.881(0.427-1.816)	0.731
ALB(g/L)( ≤35 versus >35)	0.847(0.511-1.406)	0.522	1.058(0.605-1.852)	0.843
PALB(g/L) (≤0.25 versus >0.25)	0.998(0.535-1.860)	0.994	1.752(0.738-4.157)	0.204
AFP, ng/ml (≤20 versus >20)	0.622(0.411-0.941)	0.025	0.633(0.392-1.021)	0.061
Liver cirrhosis (no versus yes)	1.067(0.638-1.784)	0.804	0.995(0.543-1.824)	0.988
Tumor size, cm (≤5 versus >5)	NA	NA	1.507(0.877-2.589)	0.138
Tumor number (single versus multiple)	1.045(0.595-1.837)	0.878	1.196(0.635-2.254)	0.580
Microvascular invasion (no versus yes)	1.650(0.981-2.775)	0.059	2.385(1.220-4.664)	0.011
Tumor encapsulation (complete versus none)	2.555(1.580-4.133)	0.000	3.485(2.060-5.896)	0.000
TNM stage (I versus II III)	2.356(1.333-4.163)	0.003	2.245(1.098-4.590)	0.027
LINC00364(low versus high)	0.576(0.379-0.876)	0.010	0.546(0.333-0.895)	0.016

Cox proportional hazards regression model.

AFP, alpha-fetoprotein; GGT, gamma glutamyl transferase; TNM, tumor-node-metastasis; ALT, alanine transaminase;

TB, total bilirubin; PALB, prealbumin; ALB, albumin; AKP, alkaline phosphatase;

HR, hazard ratio; CI, confidential interval; NA, not adopted; LINC00364, long intergenic non-coding RNA00364.

## DISCUSSION

An increasing number of long non-coding RNAs have been discovered thanks to the advances in next-generation sequencing technology [12–15, 29]. However, our understanding of their biological roles, especially in cancers, are limited. In this study, we investigated lncRNA expression profiling in response to IFN-γ treatment in HCC cells and identified a lncRNA, LncRNA00364. We found LncRNA00364 displays considerably lower expression in tumor tissue samples than in matched normal controls. Therefore, we are motivated to study the functional significance of LncRNA00364 in HCC. Overexpression of LncRNA00364 results in a range of changes which may suppress tumor formation, including proliferation inhibition, cell cycle arrest, and apoptosis induction. These results suggest that LncRNA00364 may serve as a novel tumor suppressor in HCC.

Recently, several lncRNAs have been found to play important roles in modulating tumor-suppressor and growth-arrest pathways [30, 31]. The complement of lncRNA transcription is dynamically regulated under differing cell-cycling conditions and during senescence [32, 33]. Most of expressing lncRNAs are localized in the nucleus and some of them are exported to the cytoplasm [7]. Our results showed that LncRNA00364 was mainly enriched in the cytoplasm in HCC. Cytoplasmic LncRNA00364 affects the STAT3 signaling pathway activity by inhibiting the nuclear import of phosphorylated STAT3. The accumulation of STAT3 in the nucleus is tightly controlled. A number of factors regulate the STAT3 phosphorylation and alter its nuclear import-export dynamics [34]. We found that the phosphorylation of tyrosine-705 of STAT3 was inhibited by LncRNA00364 overexpression. LncRNA00364 directly interacts with STAT3 due to the fact that LncRNA00364 shares the same conservative STAT3 transcription response element as STAT3-activated target genes. Given that inhibition of STAT3 homodimers formation diminishes STAT3 activity, STAT3 may represent a potential therapeutic target for LncRNA00364 in cancer chemotherapy.

Our results establish a link between LncRNA00364, STAT3, and IFIT2. It has been confirmed that IFIT2 is an important tumor suppressor to tumor growth, invasion, and metastasis [35–37]. Aberrant activation of STAT3 has been reported in many types of tumors. As a downstream gene of STAT3, IFIT2 displays lower expression in tumors compared to paired normal tissues. We explore how STAT3 regulate IFIT2. We find that STAT3 inhibits the transcription of IFIT2 mainly through the T705 site, mutating out of this site promotes transcription on the contrary. We further explored the functional significance of IFIT2 in HCC. IFIT2 knockdown resulted in decreased apoptosis and increased tumor growth *in vivo* and *vitro*. Our results gave evidence that IFIT2 may be a downstream target gene of LncRNA00364-STAT3, and LncRNA00364 inhibits proliferation and promotes apoptosis through STAT3-IFIT2 axis in HCC.

To explore the clinical implications of LncRNA00364, we found that LncRNA00364 level remarkably correlates with several tumor characteristics including tumor encapsulation. The lower expression of LncRNA00364 correlates with decreased survival and increased risk for postoperative recurrence in HCC patients. Univariable and multivariate analysis reveal that the LncRNA00364 expression level is an independent predictor for both OS and TTR.

**Figure 6:**
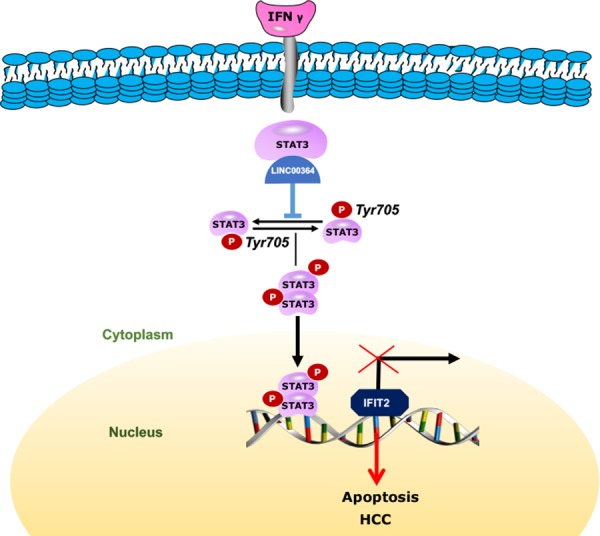
Working model shows that LncRNA00364 may contribute to the development of HCC

In summary, our study reveals that LncRNA00364 is a novel tumor suppressor and can repress HCC development by decreasing cell proliferation, inhibiting G1-S cell cycle progression and promoting apoptosis *in vitro* and *in vivo*. Mechanistically, LncRNA00364 specifically inhibits STAT3 phosphorylation by the interaction with STAT3. STAT3 inhibits the transcription of IFIT2 mainly through the T705 site. More importantly, LncRNA00364 may contribute to the development of HCC by activation of IFN-γ functions (Figure 6). Taken together, our findings revealed that the IFNγ-LncRNA00364-STAT3-IFIT2 axis exhibits exciting anticancer effects and provide new insight into a possible novel therapeutic avenue in HCC.

## MATERIALS AND METHODS

### Xenografted tumor formation in mice

Male nude mice and C57BL/6mice were purchased from SLAC Laboratory Co., Ltd (Shanghai, China) and were divided into two groups according to statistically random distribution. In one group, exponentially growing LM3 Cells, LM3-LINC00364 cells were harvested and resuspended in PBS buffer respectively. The number of 6 × 10^6^ cells in 200μl of PBS suspension were subcutaneously injected into the two sides of the back of 8-week-old nude mouse respectively (n=6 for each group). In another group, LM3-LINC00364-vector and LM3-LINC00364-ShIFIT2 cells were subcutaneously injected into the two sides of the back of nude mouse respectively. The size of the tumor was estimated according to the formula V=0.52 × L × W2 starting from 10 days after inoculation. All animal experiments were approved by the Institutional Animal Care and Use Committee of Shanghai in accordance with the National Research Council Guide for Care and Use of Laboratory Animals (SCXK [Shanghai 2007–0005]).

### Plasmids

The human LncRNA00364 was subcloned into pcDNA3.1-Flag vector with Not1 and Bamh1.

### Patients and tissue samples

We recruited 157 pair patients with primary HCC from (Liver Cancer Institute, Zhongshan Hospital, Shanghai) which tumor tissues and para-tumor tissues were obtained from 2012-2013 in this study. 57 pair fresh HCC samples and para-tumor tissues were obtained for RNA extraction and qRT-PCR analyses. Informed consents have been obtained from all patients and the project had been approved by Zhongshan Hospital Research Ethics Committee.

### Western blot and antibodies

Western blot analyses were performed as described previously. Antibodies used in these assays are as follows: pSTAT1 (Lys310) (#12629), STAT1 (4135), STAT3(#4904), pSTAT3(Tyr705) (#9145), pSTAT3(Ser727) (#9134), antibodies were purchased from Cell signaling technology. IFIT2 (sc-390724) andβ-Actin (sc-81178) antibody was purchased from Santa Cruz Biotechnology.

Stattic (ab120952) was purchased from abcam. IFN-γ(catalog #300-02) was purchased from Pepro Tech.

### Real-time PCR analysis

Total RNA was extracted from cells with TRIzol reagent (Life Technology) following the manufacturer's instructions. Complementary DNAs were synthesized with 2 μg of total RNA using iScript cDNA Synthesis Kit (Fermentas). The detection and quantification of target mRNA were performed with real-time PCR.

### Cell culture, transfection and retroviral infection

HepG2, LM3, PLC, Hep3B and 7721 were obtained from the ATCC and were tested and authenticated by karyotyping. These cells were cultured in DMEM supplemented with 10% FBS, 2mML- glutamine and penicillin (50 U/ml)/streptomycin (50 μg/ml) at 37 °C under 5% CO2 in a humidified chamber. The transfection was performed using Lipofectamine 2000 (Invitrogen, Carlsbad, CA, USA) as described. For viral infection, supernatants containing viruses were packed from 293T cells. When growing to 60–80% confluence, HepG2 and LM3 cells were infected with viral supernatants, and 5 μg/ml puromycin was added to select the stable cells. Cell cycle, cell viability and apoptosis assays were described in Supplementary Materials.

### RNA FISH analysis

HCC cells cultured on a glass coverslip were fixed in 4 % paraformaldehyde for 30 min and permeabilized with 0.1 % Triton X-100 for 30 min. The non labeled control: (5′-CCTGGTTTTTAAGGAGTGTCGCCAGAGTGCCGCGAATGAAAAA-3′). The FAM-labeled LncRNA00364 probe (5′-TCACCCTCAAGCCTGGATA-3′), which was diluted in hybridization buffer, was deposited on a surface in a humid dark chamber. The glass coverslip was then placed face down on the drop and incubated at 65 °C for 3.5hrs. After the cells were incubated with the LncRNA00364 probe. We continued with several rounds of washing (which also includes an optional DAPI staining step) and finishing with mounting the coverslip onto a micro-scope slide using an anti-fade mounting medium.

### Luciferase reporter assay

293T cells were seeded 24hrs before transfection in 24 well plates at 50%-60% confluence. The IFIT2 or truncation reporter constructs were co-transfected along with STAT3 and mutation or vector using lipofectamine 3000. After 48 hrs, luciferase activity was assessed using the Dual-Luciferase Reporter reagent following the manufacturer's instructions. Renilla luciferase was used for normalization.

### Statistical analysis

Significant association was defined when P<0.05 compared with control by using the student's t test. Pearson's correlation analysis was used to determine the correlation of the expression levels of target genes using SPSS 19.0 software.

## SUPPLEMENTARY MATERIALS FIGURES AND TABLES





## References

[1] Zhao M, Liu Y, O’Mara TA (2016). ECGene: a literature-based knowledgebase of endometrial cancer genes. Hum Mutat.

[2] Guo S, Chen W, Luo Y, Ren F, Zhong T, Rong M, Dang Y, Feng Z, Chen G (2015). Clinical implication of long non-coding RNA NEAT1 expression in hepatocellular carcinoma patients. Int J Clin Exp Pathol.

[3] Han D, Wang M, Ma N, Xu Y, Jiang Y, Gao X (2015). Long noncoding RNAs: novel players in colorectal cancer. Cancer Lett.

[4] Schmitt AM, Chang HY (2016). Long noncoding RNAs in cancer pathways. Cancer cell.

[5] Gill D, Veltkamp R (2016). Dynamics of T cell responses after stroke. Curr Opin Pharmacol.

[6] Alvarado DM, McCall K, Hecht JT, Dobbs MB, Gurnett CA (2016). Deletions of 5’ HOXC genes are associated with lower extremity malformations, including clubfoot and vertical talus. J Med Genet.

[7] Tichon A, Gil N, Lubelsky Y, Havkin Solomon T, Lemze D, Itzkovitz S, Stern-Ginossar N, Ulitsky I (2016). A conserved abundant cytoplasmic long noncoding RNA modulates repression by Pumilio proteins in human cells. Nat Commun.

[8] Kim J, Kim KM, Noh JH, Yoon JH, Abdelmohsen K, Gorospe M (2016). Long noncoding RNAs in diseases of aging. Biochim Biophys Acta.

[9] Chang YN, Zhang K, Hu ZM, Qi HX, Shi ZM, Han XH, Han YW, Hong W (2016). Hypoxia-regulated lncRNAs in cancer. Gene.

[10] Zou L, Tu G, Xie W, Wen S, Xie Q, Liu S, Li G, Gao Y, Xu H, Wang S, Xue Y, Wu B, Lv Q (2016). LncRNA NONRATT021972 involved the pathophysiologic processes mediated by P2×7 receptors in stellate ganglia after myocardial ischemic injury. Purinergic Signal.

[11] Huang MD, Chen WM, Qi FZ, Sun M, Xu TP, Ma P, Shu YQ (2015). Long non-coding RNA TUG1 is up-regulated in hepatocellular carcinoma and promotes cell growth and apoptosis by epigenetically silencing of KLF2. Mol Cancer.

[12] Cao C, Sun J, Zhang D, Guo X, Xie L, Li X, Wu D, Liu L (2015). The long intergenic noncoding RNA UFC1, a target of MicroRNA 34a, interacts with the mRNA stabilizing protein HuR to increase levels of beta-catenin in HCC cells. Gastroenterology.

[13] Chang L, Wang G, Jia T, Zhang L, Li Y, Han Y, Zhang K, Lin G, Zhang R, Li J, Wang L (2016). Armored long non-coding RNA MEG3 targeting EGFR based on recombinant MS2 bacteriophage virus-like particles against hepatocellular carcinoma. Oncotarget.

[14] Chen CL, Tseng YW, Wu JC, Chen GY, Lin KC, Hwang SM, Hu YC (2015). Suppression of hepatocellular carcinoma by baculovirus-mediated expression of long non-coding RNA PTENP1 and MicroRNA regulation. Biomaterials.

[15] Cui M, Xiao Z, Wang Y, Zheng M, Song T, Cai X, Sun B, Ye L, Zhang X (2015). Long noncoding RNA HULC modulates abnormal lipid metabolism in hepatoma cells through an miR-9-mediated RXRA signaling pathway. Cancer Res.

[16] Yuan SX, Wang J, Yang F, Tao QF, Zhang J, Wang LL, Yang Y, Liu H, Wang ZG, Xu QG, Fan J, Liu L, Sun SH, Zhou WP (2016). Long noncoding RNA DANCR increases stemness features of hepatocellular carcinoma by derepression of CTNNB1. Hepatology.

[17] Meng Z, Wang X, Gan Y, Zhang Y, Zhou H, Ness CV, Wu J, Lou G, Yu H, He C, Xu R, Huang W (2012). Deletion of IFNgamma enhances hepatocarcinogenesis in FXR knockout mice. J Hepatol.

[18] Green DS, Nunes AT, Annunziata CM, Zoon KC (2016). Monocyte and interferon based therapy for the treatment of ovarian cancer. Cytokine Growth Factor Rev.

[19] Giannopoulos A, Constantinides C, Fokaeas E, Stravodimos C, Giannopoulou M, Kyroudi A, Gounaris A (2003). The immunomodulating effect of interferon-gamma intravesical instillations in preventing bladder cancer recurrence. Clin Cancer Res.

[20] Niccolai E, Taddei A, Ricci F, Rolla S, D’Elios MM, Benagiano M, Bechi P, Bencini L, Ringressi MN, Pini A, Castiglione F, Giordano D, Satolli MA (2016). Intra-tumoral IFN-gamma-producing Th22 cells correlate with TNM staging and the worst outcomes in pancreatic cancer. Clin Sci (London).

[21] Gao J, Shi LZ, Zhao H, Chen J, Xiong L, He Q, Chen T, Roszik J, Bernatchez C, Woodman SE, Chen PL, Hwu P, Allison JP (2016). Loss of IFN-gamma pathway genes in tumor cells as a mechanism of resistance to anti-CTLA-4 therapy. Cell.

[22] Gomez JA, Wapinski OL, Yang YW, Bureau JF, Gopinath S, Monack DM, Chang HY, Brahic M, Kirkegaard K (2013). The NeST long ncRNA controls microbial susceptibility and epigenetic activation of the interferon-gamma locus. Cell.

[23] Nault JC, Fabre M, Couchy G, Pilati C, Jeannot E, Tran Van Nhieu J, Saint-Paul MC, De Muret A, Redon MJ, Buffet C, Salenave S, Balabaud C, Prevot S (2012). GNAS-activating mutations define a rare subgroup of inflammatory liver tumors characterized by STAT3 activation. J Hepatol.

[24] Yang S, Luo C, Gu Q, Xu Q, Wang G, Sun H, Qian Z, Tan Y, Qin Y, Shen Y, Xu X, Chen SH, Chan CC (2016). Activating JAK1 mutation may predict the sensitivity of JAK-STAT inhibition in hepatocellular carcinoma. Oncotarget.

[25] Lin L, Yao Z, Bhuvaneshwar K, Gusev Y, Kallakury B, Yang S, Shetty K, He AR (2014). Transcriptional regulation of STAT3 by SPTBN1 and SMAD3 in HCC through cAMP-response element-binding proteins ATF3 and CREB2. Carcinogenesis.

[26] Jia H, Song L, Cong Q, Wang J, Xu H, Chu Y, Li Q, Zhang Y, Zou X, Zhang C, Chin YE, Zhang X, Li Z (2017). The LIM protein AJUBA promotes colorectal cancer cell survival through suppression of JAK1/STAT1/IFIT2 network. Oncogene.

[27] Wang Y, Zhang L, Zheng X, Zhong W, Tian X, Yin B, Tian K, Zhang W (2016). Long non-coding RNA LINC00161 sensitises osteosarcoma cells to cisplatin-induced apoptosis by regulating the miR-645-IFIT2 axis. Cancer Lett.

[28] Lai KC, Liu CJ, Chang KW, Lee TC (2013). Depleting IFIT2 mediates atypical PKC signaling to enhance the migration and metastatic activity of oral squamous cell carcinoma cells. Oncogene.

[29] Cui M, Zheng M, Sun B, Wang Y, Ye L, Zhang X (2015). A long noncoding RNA perturbs the circadian rhythm of hepatoma cells to facilitate hepatocarcinogenesis. Neoplasia.

[30] Liu F, Yuan JH, Huang JF, Yang F, Wang TT, Ma JZ, Zhang L, Zhou CC, Wang F, Yu J, Zhou WP, Sun SH (2016). Long noncoding RNA FTX inhibits hepatocellular carcinoma proliferation and metastasis by binding MCM2 and miR-374a. Oncogene.

[31] Wang X, Sun W, Shen W, Xia M, Chen C, Xiang D, Ning B, Cui X, Li H, Li X, Ding J, Wang H (2016). Long non-coding RNA DILC regulates liver cancer stem cells via IL-6/STAT3 axis. J Hepatol.

[32] Ge Y, Yan X, Jin Y, Yang X, Yu X, Zhou L, Han S, Yuan Q, Yang M (2015). MiRNA-192 [corrected] and miRNA-204 directly suppress lncRNA HOTTIP and interrupt GLS1-mediated glutaminolysis in hepatocellular carcinoma. PLoS Genet.

[33] George J, Patel T (2015). Noncoding RNA as therapeutic targets for hepatocellular carcinoma. Semin Liver Dis.

[34] Guo W, Liu S, Cheng Y, Lu L, Shi J, Xu G, Li N, Cheng K, Wu M, Cheng S, Liu S (2016). ICAM-1-related noncoding RNA in cancer stem cells maintains ICAM-1 expression in hepatocellular carcinoma. Clin Cancer Res.

[35] Reich NC (2013). A death-promoting role for ISG54/IFIT2. J Interferon Cytokine Research.

[36] Stawowczyk M, Van Scoy S, Kumar KP, Reich NC (2011). The interferon stimulated gene 54 promotes apoptosis. J Biol Chem.

[37] Diamond MS, Farzan M (2013). The broad-spectrum antiviral functions of IFIT and IFITM proteins. Nat Rev Immunol.

