# Supplementary material for: Long non-coding RNA00364 represses hepatocellular carcinoma cell proliferation via modulating p-STAT3-IFIT2 signaling axis
Source: Oncotarget. 2017 Oct 25;8(60):102006–19. doi: 10.18632/oncotarget.22039 (PMC5731931; doi:10.18632/oncotarget.22039)
Supplement: Supplementary file 1 [file oncotarget-08-102006-s001.pdf]

# Long non-coding RNA00364 represses hepatocellular carcinoma cell proliferation via modulating p-STAT3-IFIT2 signaling axis

## SUPPLEMENTARY MATERIALS

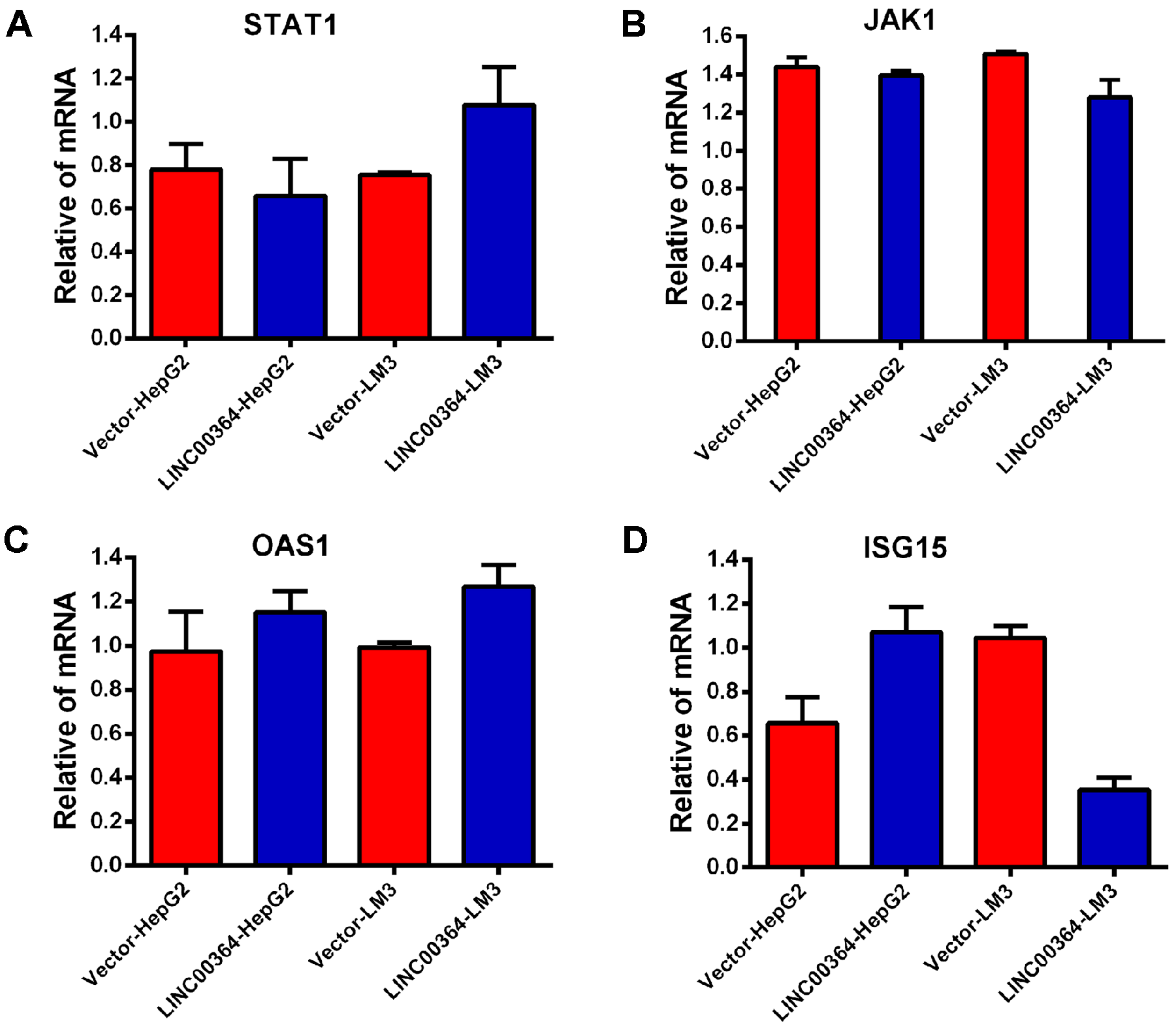


**Supplementary Figure 1:** The mRNA level of STAT1 **(A)**, JAK1 **(B)**, OAS1 **(C)** and ISG15 **(D)** analyzed by Real-time PCR assays.

**Supplementary Table 1: The upregulated genes induced by interferon–γ treatment based on microarray analysis.**

**See Supplememtary File 1**
