# Supplementary material for: Long non-coding RNA00364 represses hepatocellular carcinoma cell proliferation via modulating p-STAT3-IFIT2 signaling axis
Source: Oncotarget. 2017 Oct 25;8(60):102006–19. doi: 10.18632/oncotarget.22039 (PMC5731931; doi:10.18632/oncotarget.22039)
Supplement: Supplementary file 2 [file oncotarget-08-102006-s002.docx]

1. Homo sapiens long intergenic non-protein coding RNA 364 (LINC00364), non-coding RNA.
2. Homo sapiens uncharacterized LOC 143666 (LOC143666), non-coding RNA.
3. Homo sapiens long intergenic non-protein coding RNA 899 (LINC00899), non-coding RNA.
4. Homo sapiens long intergenic non-protein coding RNA 116 (LINC00116), non-coding RNA.
5. Homo sapiens uncharacterized LOC202781 (LOC202781), non-coding RNA.
6. Homo sapiens long intergenic non-protein coding RNA 942 (LINC00942), non-coding RNA.
7. Homo sapiens uncharacterized LOC200772 (LOC200772), non-coding RNA.
8. Homo sapiens small nucleolar RNA host gene 8 (non-protein coding) (SNHG8), transcript variant 2, non-coding RNA.
9. Homo sapiens uncharacterized LOC79015 (LOC79015), non-coding RNA.
10. Homo sapiens uncharacterized LOC100499405 (LOC1000499405), non-coding RNA.
11. Homo sapiens uncharacterized LOC644656 (LOC644656), non-coding RNA.
12. Homo sapiens uncharacterized LOC100506548 (LOC100506548), non-coding RNA.
13. Homo sapiens MIR 210 host gene (non-protein coding) (MIR210HG), non-coding RNA.
14. Homo sapiens long intergenic non-protein coding RNA 707 (LINC00707), non-coding RNA.
15. Homo sapiens uncharacterized LOC643401 (LOC643401), non-coding RNA.
16. Homo sapiens uncharacterized LOC100505633 (LOC100505633), non-coding RNA.
17. Homo sapiens uncharacterized LOC100287015 (LOC100287015), non-coding RNA.
